# 153. Utilization of Post-Exposure Prophylaxis to Prevent Lyme Disease in a Large US Healthcare Database

**DOI:** 10.1093/ofid/ofab466.355

**Published:** 2021-12-04

**Authors:** Grace E Marx, Candace C Fuller, Nicole Haug, Dave Martin, Catherine Corey, Alyssa Beck, Amy M Schwartz, Alison F Hinckley

**Affiliations:** 1 Centers for Disease Control and Prevention, Fort Collins, Colorado; 2 Harvard Medical School, Harvard Pilgrim Health Care Institute, Boston, Massachusetts; 3 StatLog Econometrics, Inc., Québec City, Quebec, Canada; 4 US Food and Drug Administration, Silver Spring, Maryland

## Abstract

**Background:**

In the United States, at least 50,000 emergency department visits for tick bite and an estimated 476,000 Lyme disease diagnoses occur annually, with incidence of both high among children. The majority of these healthcare visits occur in the northeastern and midwestern states having high Lyme disease incidence and during the summer and fall months, corresponding to peak opportunities for exposure to blacklegged ticks. Post-exposure prophylaxis (PEP) with a single dose of doxycycline can effectively prevent Lyme disease after a tick bite that is high risk for transmission of Lyme disease. We describe characteristics of patients with dispensings of single-dose doxycycline in a large US-based system that includes patients enrolled in private and public health insurance plans.

**Methods:**

Single-dose doxycycline (≤200 mg) dispensings during January 2009 – February 2020 were identified for patients enrolled in seven Data Partners that contributed electronic healthcare data to the Food and Drug Administration Sentinel Distributed Database, including large national insurers, an integrated delivery care network, a state Medicaid, and the 100% Medicare fee-for-service plan. We examined patient and PEP dispensing characteristics by patient age, state of residence, and month of dispensing.

**Results:**

We identified 408,897 patients with PEP (n=474,414 total dispensings) with a mean age of 60 years at first dispensing. Overall, there were 21 patients per 10,000 eligible members with PEP dispensings. Dispensings were less common in children (< 1 and 4 patients per 10,000 eligible members aged < 8 and 8-18 years, respectively). Most dispensings (72%) occurred in states with high incidence of Lyme disease. Seasonality of dispensings was bimodal, with most occurring during April – July and October – November (71 – 83%, by year).

**Conclusion:**

Lyme disease PEP was relatively common and mirrored geographic and seasonal trends observed for ED visits for tick bites and Lyme disease diagnoses. However, we observed more PEP among older adults, and few dispensings among children. Despite healthcare visits for tick bites and Lyme disease occurring disproportionately among pediatric age groups, PEP appears to be underutilized in children.

**Disclosures:**

**All Authors**: No reported disclosures

